# Production of Superoxide in Bacteria Is Stress- and Cell State-Dependent: A Gating-Optimized Flow Cytometry Method that Minimizes ROS Measurement Artifacts with Fluorescent Dyes

**DOI:** 10.3389/fmicb.2017.00459

**Published:** 2017-03-21

**Authors:** Megan E. McBee, Yok H. Chionh, Mariam L. Sharaf, Peiying Ho, Maggie W. L. Cai, Peter C. Dedon

**Affiliations:** ^1^Infectious Disease Interdisciplinary Research Group, Singapore-MIT Alliance for Research and TechnologySingapore, Singapore; ^2^Department of Microbiology and Immunology, Yong Loo Lin School of Medicine, National University of SingaporeSingapore, Singapore; ^3^Department of Biological Engineering, Massachusetts Institute of Technology, CambridgeMA, USA

**Keywords:** reactive oxygen species (ROS), flow cytometry, bacteria, oxidative stress, starvation, antibiotics

## Abstract

The role of reactive oxygen species (ROS) in microbial metabolism and stress response has emerged as a major theme in microbiology and infectious disease. Reactive fluorescent dyes have the potential to advance the study of ROS in the complex intracellular environment, especially for high-content and high-throughput analyses. However, current dye-based approaches to measuring intracellular ROS have the potential for significant artifacts. Here, we describe a robust platform for flow cytometric quantification of ROS in bacteria using fluorescent dyes, with ROS measurements in 10s-of-1000s of individual cells under a variety of conditions. False positives and variability among sample types (e.g., bacterial species, stress conditions) are reduced with a flexible four-step gating scheme that accounts for side- and forward-scattered light (morphological changes), background fluorescence, DNA content, and dye uptake to identify cells producing ROS. Using CellROX Green dye with *Escherichia coli, Mycobacterium smegmatis*, and *Mycobacterium bovis* BCG as diverse model bacteria, we show that (1) the generation of a quantifiable CellROX Green signal for superoxide, but not hydrogen peroxide-induced hydroxyl radicals, validates this dye as a superoxide detector; (2) the level of dye-detectable superoxide does not correlate with cytotoxicity or antibiotic sensitivity; (3) the non-replicating, antibiotic tolerant state of nutrient-deprived mycobacteria is associated with high levels of superoxide; and (4) antibiotic-induced production of superoxide is idiosyncratic with regard to both the species and the physiological state of the bacteria. We also show that the gating method is applicable to other fluorescent indicator dyes, such as the 5-carboxyfluorescein diacetate acetoxymethyl ester and 5-cyano-2,3-ditolyl tetrazolium chloride for cellular esterase and reductive respiratory activities, respectively. These results demonstrate that properly controlled flow cytometry coupled with fluorescent probes provides precise and accurate quantitative analysis of ROS generation and metabolic changes in stressed bacteria.

## Introduction

Reactive oxygen species (ROS) have been proposed to play critical roles in microbial metabolism (Imlay, 2013) and response to environmental stressors such as antibiotic exposure (Brynildsen et al., 2013; Zhao and Drlica, 2014). Though “ROS” are often viewed as a single agent, the widely differing chemistry of specific types of ROS critically determines their function in microbes. Among the biologically relevant ROS, superoxide (O_2_^•–^) is a relatively unreactive species (t_1/2_ ∼ 1–15 min) arising from electron transport processes and cytochrome activity, and is detoxified by superoxide dismutases (SOD) to the relatively inert hydrogen peroxide (H_2_O_2_) (Hassan and Fridovich, 1979; Iyanagi, 1990; Dedon and Tannenbaum, 2004). Reduction of H_2_O_2_ by redox-active metals (e.g., Fe^2+^/Fe^3+^ and Cu^1+^/Cu^2+^ in Fenton chemistry), yields highly reactive hydroxyl radicals (t_1/2_ ∼ 10^-9^ s) (Imlay et al., 1988; Imlay, 2013, 2015; Bataineh et al., 2016). Though less abundant in bacteria, another source of hydroxyl radicals involves homolytic scission of peroxynitrite (ONOO^-^; t_1/2_ ∼ 1 s) arising from the reaction of nitric oxide (NO) and O_2_^•–^ (Dedon and Tannenbaum, 2004).

The recent emergence of fluorescent probes to detect ROS builds on the decades-long use of fluorescent dyes to study bacterial components, such as nucleic acid abundance or dehydrogenase activity (Walberg et al., 1999; Nebe-von-Caron et al., 2000). In bacteria, fluorescent probes have been most widely used in attempts to define the role of ROS production in response to antibiotic exposures (Albesa et al., 2004; Goswami et al., 2006; Kohanski et al., 2007; Wang et al., 2010; Dwyer et al., 2012). A variety of fluorescent ROS probes are now available, with each probe claiming to possess different reactivity, stability, fluorescence and membrane transport properties (Kalyanaraman et al., 2012; Imlay, 2015). However, the efficiency and specificity of different dyes for detecting different ROS *in vivo* have rarely been established with any confidence (Kalyanaraman et al., 2012), much less across a range of different bacteria. Among other dyes, bacterial ROS have often been studied using 3′-(*p*-hydroxyphenyl) fluorescein (HPF) (Kohanski et al., 2007; Dwyer et al., 2012, 2014) and 5- or 6-chloromethyl-2′,7′-dichlorodihydrofluorescein diacetate (CM-H_2_DCFDA) (Roca and Ramakrishnan, 2013; Dwyer et al., 2014). The latter is prone to signal artifacts due to erroneous amplification of the fluorescence intensity by a redox-cycling mechanism involving an intermediate radical (Kalyanaraman et al., 2012). This problem has led several groups to use HPF for ROS analysis in bacteria (Kohanski et al., 2007; Nguyen et al., 2011; Dwyer et al., 2012; Grant et al., 2012), but HPF almost exclusively detects “highly ROS” such as hydroxyl radical and hypochlorous acid (Setsukinai et al., 2003). While there is emerging evidence for involvement of ROS in antibiotic-induced stress in a limited range of model bacteria, mainly *Escherichia coli*, recent studies have challenged the generality of antibiotic-induced ROS production on the basis of the low dynamic range of HPF (Renggli et al., 2013) and problems with spurious fluorescent signals and other artifacts (Keren et al., 2013; Liu and Imlay, 2013; Renggli et al., 2013; Paulander et al., 2014).

There are other factors that complicate the use of ROS-sensitive dyes. Among these are the uptake and removal of both unreacted and ROS-activated dye molecules from the cells by efflux pumps, increased membrane permeability caused by cellular stress (Renggli et al., 2013), as well as poorly defined bacterial metabolic states at the time of the stress and dye staining. For example, while *E. coli* does not possess cytochrome P450s, mycobacteria have dozens (McLean et al., 2010; Ouellet et al., 2010), with P450s as well-established sources of O_2_^•–^ (Munro et al., 2013). The difficulty of measuring ROS coupled with these idiosyncratic factors in individual bacteria and across different bacterial species have hindered a broader examination of cell state as a factor in stress-induced ROS generation and its role in the decision between cell death and survival.

To overcome these problems, we developed a robust, artifact-minimized platform for high-throughput flow cytometric detection of ROS in bacteria using a stable, DNA-binding fluorescent dye. This standardized approach employs a four-step gating scheme to account for side-scattered and forward-scattered light, DNA content, and dye uptake to accurately identify and quantify ROS-producing cells. The method accounts for autofluorescence arising from changes in cell morphology, membrane composition, and protein expression during normal cell growth and the cellular response to stresses such as antibiotic treatment and starvation (Kim et al., 2011; Renggli et al., 2013; Paulander et al., 2014).

We piloted this method with CellROX Green dye to assess its activity in living bacteria and to assess claims (Choi et al., 2015) that CellROX Green is selective for O_2_^•–^ in bacterial cells. There are many methods for detecting O_2_^•–^, including electrochemistry, UV-VIS spectroscopy, chemiluminescence, and vibrational spectroscopy (Hayyan et al., 2016), though intracellular O_2_^•–^ has traditionally been quantified by extracellular reduction of ferricytochrome c or by electron spin-resonance (ESR) with spin trapping by 5-diethoxyphosphoryl-5-methyl-1-pyrroline *N*-oxide (Frejaville et al., 1995; Huycke et al., 1996; Korshunov and Imlay, 2006). Neither of these bulk measurements provide single-cell resolution of endogenous O_2_^•–^ in bacterial populations. Though new probes specific to O_2_^•–^ and suitable for direct *in vivo* studies have been developed in recent years, they have not been evaluated in bacteria (Mukhopadhyay et al., 2007; Hu et al., 2015; Shin et al., 2015). Here, we applied our flow cytometric method with CellROX Green to quantify O_2_^•–^ production in three diverse but well-studied model bacteria, *E. coli* and the mycobacterial species *Mycobacterium smegmatis* and *Mycobacterium bovis* bacille Calmette-Guérin (BCG), all subjected to the well-studied stresses of antibiotic exposure and nutrient depletion. We found that changes in O_2_^•–^ production across isogenic bacterial populations are heterogeneous and highly dependent upon the nature of the stress and the physiological state of the cells. We also demonstrate the utility of the flow-cytometry method for two other metabolism-sensitive fluorescent dyes: 5-carboxyfluorescein diacetate acetoxymethyl ester (CFDA) for esterase activity (Schreer et al., 2005) and 5-cyano-2,3-ditolyl tetrazolium chloride (CTC) for cellular respiratory activity (Rodriguez et al., 1992).

## Materials and Methods

### Bacteria Strains and Culture

*Mycobacterium smegmatis* mc^2^155 (ATCC 700084) was plated on 7H10 agar supplemented with 10% Middlebrook Oleic Albumin Dextrose Catalase (OADC) growth supplement and 0.2% glycerol. After picking three separate colonies, the mycobacterial strain was grown in 10 mL of 7H9 media supplemented with 10% albumin D-glucose-salt supplement (ADS), 0.2% glycerol, 0.05% Tween 80 at 37°C in a shaker. *M. smegmatis* was passaged twice before treatment of mid-log growing cells. *M. bovis* bacille Calmette Guérin (BCG) Pasteur strain 1172P2 was cultured from isogenic freezer stocks in 50 mL conical tubes then passaged into roller bottles with supplemented 7H9 media (Chionh et al., 2016). For nutrient-deprived (i.e., starved) cultures, *M. smegmatis* and BCG were grown to OD_600_ 0.8–1.0, washed twice in PBS (137 mM NaCl, 2.7 mM KCl, 10 mM NaH_2_PO_4_, 1.8 mM KH_2_PO_4_, pH 7.4), and re-suspended to an OD_600_ ∼1.0 (BCG) or 0.1 (*M. smegmatis*) in PBS plus 0.05% Tyloxapol (non-metabolizable detergent to prevent clumping) in roller bottles. CFUs for BCG were determined from 10-fold serial dilution plating on 7H10 at 37°C for 14–21 days. Single colonies of *E. coli* DH5α or K-12 BW25113 were grown overnight in LB broth, diluted 1:100 in fresh LB as a subculture and grown to OD_600_ 0.4–0.6 to use in studies. CFUs for *E. coli* and *M. smegmatis* was determined from 10-fold serial dilution plating on LB incubated overnight (*E. coli*) or 2 days (*M. smegmatis*) at 37°C. Knock-out mutants for *sodA* and *sodB* in the *E. coli* K-12 BW25113 background were obtained from the *E. coli* Keio Knockout Collection (GE Dharmacon).

### Antibiotic and Toxicant Exposures

Antibiotics were subjected to twofold serial dilutions in 7H9 media or PBS in 96-well plates containing 100 μL per well. For H_2_O_2_ and menadione treatments, reagents were added to each well of a 96-well plate at concentrations twofold higher than the final exposure condition. Bacterial cultures were diluted to an OD 0.1 (*M. smegmatis* and *E. coli*) or 0.2 (BCG) and 100 μL was added to each well containing antibiotic, H_2_O_2_, menadione, media or PBS, for a total treatment volume of 200 μL. The plates were incubated for indicated times at 37°C. For menadione and H_2_O_2_ treatments, cultures were washed once and resuspended in PBS, while antibiotic-treated cells were diluted in fresh media without washing. For the PBS versus 10% 7H9/LB, 20 uL of culture was transferred directly (no wash) into either 180 uL of 100% PBS or PBS + 10% LB/7H9 in the presence of 0.5 uM CellROX (a 10x dilution). After 30 min cells were fixed by the addition of paraformaldehyde (PFA; 4%) and analyzed by flow cytometry. Antibiotic-tolerant *M. smegmatis* were generated by diluting mid-log growing cells to OD 0.1 and aliquoting 100 μL to individual wells containing an antibiotic at a range of concentrations in a 96-well plate. Cultures were covered and incubated at 37°C with humidity for 1 or 5 days prior to analysis.

### Flow Cytometry and Dye Staining

For flow cytometry staining, 20 μL volumes of treated cells were added to 180 μL of 0.5 μM CellROX Green (ThermoFisher), 5 mM CTC (ThermoFisher), or 10 ng/mL CFDA (ThermoFisher) in PBS in 96-well V-bottom plates and incubated for 30 min (1 h for CTC) at 37°C. For menadione and H_2_O_2_ treatments, CellROX Green was included in the PBS during treatment (60 min). After incubation, 50 μL of DAPI (5 μg/mL) in 4% PFA-PBS was added to the cells for >10 min at 25°C prior to analysis. Samples were analyzed within 2 h on a custom LSR II flow cytometer (BD Biosciences) with CellROX Green, CFDA, and CTC excited by an argon laser and detected with a 530/30 nm band-pass emission filter or 670 LP emission filter for CTC. DAPI was excited with a UV laser and detected with a 450/50 nm band-pass emission filter. Samples were analyzed using a HTS fluidics system directly from the 96-well V-bottom staining plate. The flow rate was set to 3.0 μL/s with a 150 μL injection volume, 100 μL mixing volume, 250 μL/s mixing speed, five mixes, and a wash volume of 800 μL. For each sample 50,000–200,000 events were collected when possible. For antibiotic treatments, only samples with >10,000 DAPI^+^ cells were analyzed. Data was collected by Diva (BD Biosciences) and analyzed with FlowJo v10.0.6 (Tree Star, Inc.). Gating was set using unstained samples for the bacterial population by forward-scatter (FSC; correlates with cell size) and side-scatter (SSC; correlates with cell internal granularity) of light and to determine background fluorescence. Single DAPI-stained cells were used to gate the DNA-positive population, while single CellROX Green-, CFDA-, or CTC-stained cells were used to determine the dye-positive population. The single-color stains were also used for compensation, as needed, between the dye signals. A Biotek Synergy 4 microplate reader was used for fluorescence measurements with excitation of CellROX Green using a 485/20 nm filter and emission captured at 528/20 nm. Statistical analyses were performed with Prism GraphPad Software 6.

## Results

### Design Criteria for an Artifact-Minimized Method for High-Throughput Quantification of Bacterial ROS Using Fluorescent Dyes

**FIGURE 1:**
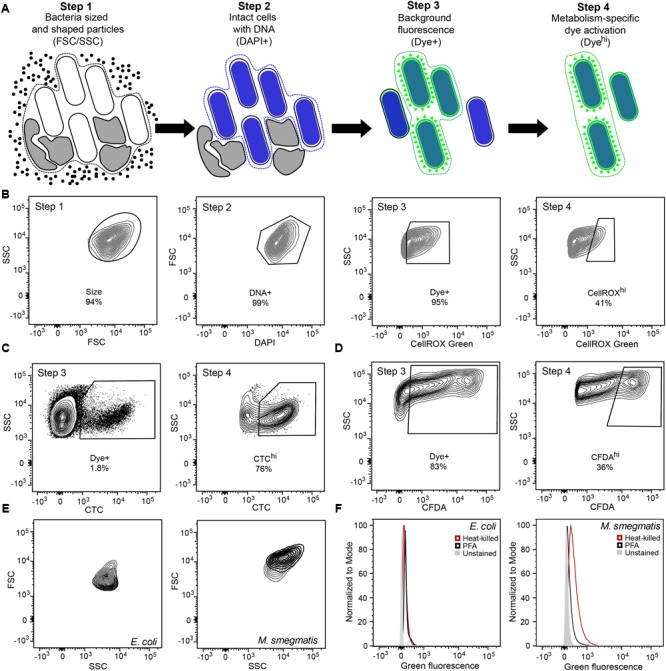
**Optimization of gating and other flow cytometric parameters minimizes artifacts in reactive oxygen species (ROS) detection with fluorescent probes in bacteria. (A)** Workflow diagram summarizing the four-step flow cytometry gating strategy. *Step 1* selects for bacteria-sized and -shaped particles. *Step 2* excludes particles that do not contain DNA. *Step 3* accounts for background fluorescence by eliminating cells that did not take up the dye and cells with non-specific autofluorescence. *Step 4* quantifies metabolism-associated dye activation. The gating process and rationale is further elaborated in Section “Results and Discussion.” **(B)** Representative contour plots of the four-step gating scheme *of Mycobacterium smegmatis* stained with DAPI and the ROS-reactive dye CellROX Green. Cells are sequentially selected for FSC and SSC (*Size*), DAPI staining (*DNA^+^*), dye presence (*Dye^+^*), and dye reactivity (*CellROX^hi^*). Representative contour plots of *Steps 3 and 4* for CTC **(C)** and CFDA **(D)** analyses in *Mycobacterium bovis* BCG. **(E)** SSC and FSC contour plots of log-growing (black) and antibiotic-exposed (gray) *Escherichia coli* and *M. smegmatis*. *E. coli* cultures were treated with 2 μg/mL ampicillin for 1 h and *M. smegmatis* with 2 μg/mL ethambutol for 1 h. **(F)** Histograms of DAPI-stained (DNA^+^) *E. coli* and *M. smegmatis* demonstrating increased background fluorescence in dead bacteria (heat-killed, red; PFA fixed, black) in comparison to live bacteria (gray) in the CellROX Green detection channel (green fluorescence).

The availability of fluorescent dyes for the detection and quantification of reactive chemical species in cells has fostered the development of single-cell, multi-parameter, high-throughput analyses in microbiology and antibiotic pharmacology, including both multi-well plate-based population assays and flow cytometry assays. However, factors such as dye uptake efficiency, dye efflux, changes in cell morphology, and background fluorescence complicate the application of these fluorescent dye-based assays to bacteria and confound the interpretation of observed changes in fluorescence. To achieve assay precision and accuracy and ensure credible and biologically meaningful results, we developed a four-step gating scheme (**Figures 1A,B**) to select for: (1) particle size based on FSC and SSC as light passes through the cells, (2) DNA content based on DAPI staining (or other DNA-binding dyes), (3) the presence of dye based on fluorescence above unstained and DAPI-stained particles, and (4) dye reactivity above baseline or basal fluorescence. Application of this two-dye, multi-gating flow cytometry method distinguishes bacteria from debris by selecting for size and DNA content, which also distinguishes single cells from dividing cells and individual bacterial species in mixed cultures. Additionally, greater sensitivity and specificity for ROS and metabolic activity is achieved by removing contributions from autofluorescence and by selecting only cells that have taken up the dye, with sub-gating of those cells for highly fluorescent activated dye. Here, we illustrate this scheme with CellROX^hi^ gating for endogenous ROS quantification (**Figure 1B**) and then demonstrate the versatility of the assay with examples using CTC and CFDA dyes for cellular metabolism (**Figures 1C,D**).

### A 4-Steps Gating Scheme for the Accurate Quantification of ROS Levels in Bacteria

*Step 1* in the analysis scheme (**Figures 1A,B**) defines the bacterial population of interest based on FSC and SSC properties. In addition, *Step 1* gating can be adjusted for populations in which a significant portion exhibits changes in cell morphology. By considering increases in fluorescence observed in cells with elevated SSC or FSC post-treatment, which can be performed by two-dimensional gating such as SSC versus fluorescence, these cells can be selected for analysis if they pass the subsequent gates for non-specific background fluorescence (to be considered Dye^+^) and then for desired activation of the fluorescent dye. Each bacterial cell has morphological and biological properties that may be uniquely altered in response to stress or as part of its life cycle. Gating (i.e., restricting) SSC and FSC in *Step 1* allows consistent selection for cell size, shape, complexity, and composition. For example, during logarithmic growth, SSC and FSC profiles of rod-shaped filamentous *E. coli* differ from those of *M. smegmatis* bacilli (**Figure 1E**). Overall, both bacilli and filamentous bacteria have higher FSC signals during cell division. At the same time, population heterogeneity in cell size or length and cellular macromolecular composition can be detected by FSC and SSC, respectively. For *M. smegmatis*, heterogeneity is likely due to bacterial replication on the basis of time rather than cell size (Aldridge et al., 2012). Depending upon cytometer settings, non-coccoid bacteria passing through the light source at different angles will be detected as lower or higher FSC/SSC signals than those observed for uniform spheres. Additionally, stresses such as antibiotic exposures can alter cell light scatter by inhibiting replication or altering cell morphology, such as by causing filamentation or by inhibiting septation (**Figure 1E**) (Renggli et al., 2013; Paulander et al., 2014; Saint-Ruf et al., 2016). These differences in morphology and composition detected by FSC and SSC should be considered when interpreting bacterial populations during an analysis since these changes can significantly alter the observed fluorescence.

*Step 2* ensures that only cells with intact DNA are included in the analysis. Small non-viable particles such as cell debris and dust particles also generate FSC and SSC signals. Therefore, DNA content staining is used to differentiate bacteria from particles. This staining step further enables the assessment of several biologically relevant parameters including detection of doubling cells, cell aggregates, and dead cells with fragmented DNA (Nebe-von-Caron et al., 2000). The gating also excludes artifacts from the analysis, which may arise from an abundance of dead cell debris in studies of bacterial persistence and dormancy, as pursued here with *M. smegmatis*. For example, subsequent experiments (illustrated in **Figures 4**, **5**) show that >90% of the mycobacteria were killed by antibiotics before a drug-resistant population was established, with the debris potentially confounding flow cytometry analysis.

*Step 3* accounts for dye-based background fluorescence and helps to distinguish cells that take up dye molecules from those that do not, thus increasing the accuracy and rigor of the ROS analysis. Here, the relatively weak but detectable fluorescence of unactivated dye is exploited to select all presumptive dye-containing cells by virtue of their fluorescence above that of unstained cells. This fluorescence of unactivated CellROX Green is apparent as a 10-fold increase in fluorescence in PBS solutions above the PBS background (28 versus 254 f.u. under conditions used in the flow studies) and by a >1.5-fold increase in fluorescence when CellROX Green is added to heat-killed or PFA-fixed cells (depending upon fluorimeter settings and cell concentration). It is important to note that flow cytometry cannot be used to accurately attribute the source of increased fluorescence (above unstained controls) to specific changes in cellular morphology, composition and metabolism, steady-state ROS generation, or increased dye uptake. However, using this gate for dye uptake increases the accuracy for ROS-specific events in the assay. In the absence of this step, all cells from *Steps 1* and *2* will be analyzed regardless of dye uptake status. Only after gating for size, DNA, and dye presence is it possible to accurately identify cells in which ROS has potentially activated the dye molecules to fluoresce above background levels and to quantify the fluorescence intensities of these cells.

It is important to point out that *Step 3* also accounts for changes in background fluorescence from a variety of sources. For example, in our hands, the exponential growth in the presence of LB or 7H9 cell culture medium during staining of *E. coli* and *M. smegmatis*, respectively, increased background signals by up to 20-fold. Increases in fluorescence were also observed in PFA-fixed and heat-killed bacteria (**Figure 1F**). As dead cells do not have ROS-generating metabolic processes, such fluorescence increases indicate that ungated fluorescent signals cannot be entirely attributed to the presence of ROS. In these cases, variation in the background fluorescence makes it difficult to determine whether the summed fluorescence increment, above that observed in unstained cells, is due to activated dye that reacted with ROS produced in metabolically active cells, by unreacted dye taken up by cells, or by fluorescent intracellular compounds, such as flavins (Benson et al., 1979). The Dye^+/-^ gating in *Step 3* minimizes this issue by pre-selecting cells that possess fluorescence greater than unstained cells, which includes cells that have internalized dye molecules regardless of their activation state.

In *Step 4*, the degree of dye activation, presumably by ROS, is quantified, here as “CellROX^hi^.” *Step 3* selects for cells with fluorescence above untreated or control levels, with those with higher levels of fluorescence (CellROX^hi^) are deemed to contain elevated levels of the dye-reactive chemicals, presumably ROS; the identity of the reactive chemicals as specific forms of ROS must be defined by appropriate control experiments. Heat-killed or fixed cells that take up dye, but do not produce ROS or other reactive chemicals, are used as gating controls to set the threshold for CellROX^hi^. Alternatively, ROS production above basal levels or production observed in a control population (e.g., log growing, wild-type, or untreated cells) may also be used to set the CellROX^hi^ threshold. With the prior gating steps, the normalizing denominator for the CellROX^hi^ population is the sum total of cells with detectable fluorescence rather than all bacteria analyzed in the sample.

The general applicability and utility of the gating scheme is further illustrated in BCG for reductive respiratory activities using CTC and for cellular esterase activity using CFDA-AM (**Figures 1B,C**). Similar considerations were made for FSC and SSC (*Step 1*), DNA counterstaining to differentiate cells from debris (*Step 2*), dye update and retention (*Step 3*) and dye activation above background fluorescence (*Step 4*). It is important to note here that CTC becomes insoluble upon reaction, which results in strong fluorescence from activated dye molecules retained within the cells, while the fluorescent carboxyfluoroscein product of CFDA-AM is negatively charged and thus retained within cells longer than fluorescein. This positive attribute is similar to the DNA-binding activity of ROS-activated CellRox Green.

### CellROX Green is a Sensitive Detector of Superoxide but not H_2_O_2_-Induced ROS

In addition to quantifying ROS, identifying the ROS species generated during stress is a crucial step in establishing the precise molecular mechanisms underlying the stress response. The challenge here is that the reactivity of individual fluorescent probes for specific ROS has not been clearly defined under biological conditions and many probes react with a variety of oxidants and free radical species. We chose to use CellROX Green as an ROS probe due to its unique feature of having high fluorescence quantum yields only upon ROS-activation and binding to dsDNA (Invitrogen, 2012). This feature obviates artifacts due to altered membrane integrity resulting from stress or cell state, and enhances accumulation of ROS-activated dye to maximize fluorescence signal intensity above background and autofluorescence in cells – critical to setting the Dye^+^ gate in *Step 3*. However, for this probe especially, there is a lack of clarity in the scientific community about its intracellular ROS specificity (ResearchGate, 2013; Choi et al., 2015), and, to our knowledge, there is no consensus on its reactivity in bacterial cells. CellROX Green is a propriety photostable dye, which, according to the manufacturer, exhibits ROS reactivity that complements HPF. Unlike HPF, CellROX Green reacts with hydroxyl radicals, O_2_^•–^ and *t*-butylhydroperoxide *in vitro*, but not with H_2_O_2_, NO, ONOO^-^, or HOCl (Invitrogen, 2010). However, CellROX Green has recently been claimed to react almost exclusively with O_2_^•–^ *in vivo* (Choi et al., 2015). Hence, we sought to confirm the identity of the intracellular ROS detected by CellROX Green in bacteria using two well-studied ROS generators: (1) menadione, a quinone that undergoes catalytic one-electron redox cycling to reduce molecular oxygen (O_2_) to O_2_^•–^ (t_1/2_ ∼ 1–15 min) (Hassan and Fridovich, 1979; Iyanagi, 1990), and (2) H_2_O_2_ that reacts with redox-active metals to produce extremely short-lived ferryl or hydroxyl radicals (t_1/2_ ∼ 10^-9^ s) under specific conditions (Imlay et al., 1988; Imlay, 2013, 2015; Bataineh et al., 2016).

**FIGURE 2:**
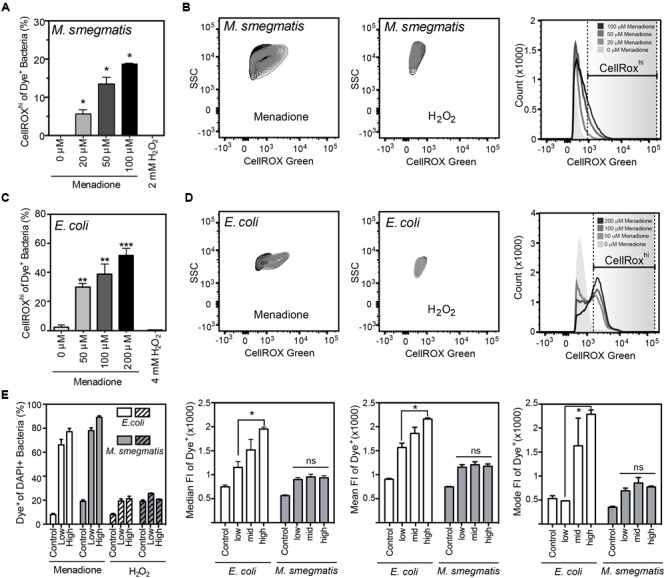
**Reactive oxygen species production detected by CellROX Green in *E. coli* and *M. smegmatis.* (A)** Proportion of *M. smegmatis* cells producing ROS after 1.5 h of treatment with menadione or H_2_O_2_. Mean ± SEM for *N* = 3. **(B)** Representative contour plots of DAPI-stained (Dye^+^) *M. smegmatis* treated (gray) with 100 μM menadione or 2 mM H_2_O_2_ or untreated (black) for 1.5 h. Representative histograms of dose-dependent increases in CellROX Green fluorescence of Dye^+^ *M. smegmatis.* **(C)** Proportion of *E. coli* (BW25113) cells producing ROS after 1 h treatment with menadione or H_2_O_2_. Mean ± SEM for *N* = 3. **(D)** Representative contour plots of Dye^+^ *E. coli* treated with 200 μM menadione (gray) or 4 mM H_2_O_2_ (gray) or untreated (black) for 1 h. Representative histograms of dose-dependent increase in CellROX Green fluorescence of Dye^+^ *E. coli.* **(E)** Changes in proportion of Dye^+^ cells during menadione or H_2_O_2_ stress in *E. coli* and *M. smegmatis* (left). Median, mean, and mode fluorescence intensity of Dye^+^ *E. coli* or *M. smegmatis* treated with menadione reflect the differential responses of the two bacteria to menadione. One-way ANOVA with Dunn’s multiple comparisons versus untreated, ^∗^*P* < 0.05, ^∗∗^*P* < 0.01, ^∗∗∗^*P* < 0.001 **(A,C)** or with Tukey’s multiple comparisons across all treatments, ^∗^*P* < 0.05 **(E)**.

CellROX Green reproducibly detected of intracellular ROS when incubated for 1 h with live bacteria in the presence of menadione in PBS (**Figure 2**). While menadione can induce CellROX Green-reactive species by indirect mechanisms, such as alterations of cell metabolism that lead to ROS production (Marinho et al., 2014), we observed no shift in FSC, SSC, or fluorescence signals in either the DAPI or CellROX Green channels (Supplementary Figures S1A,B), which ruled out menadione-induced changes in cell biochemistry or morphology that could cause increased non-specific fluorescence or light scattering. Using the four-step gating protocol to minimize signals due to menadione-induced cell permeability changes, non-cytotoxic menadione exposures (Supplementary Figure S2A) generated CellROX Green-detectable ROS in both *E. coli* and *M. smegmatis* in a dose-dependent manner (**Figures 2A–E**). Although ROS levels were not elevated in all menadione-exposed cells in an isogenic population when compared with untreated cells, both the cellular fluorescence intensity and number of cells with detectable ROS increased in a dose-dependent manner (**Figures 2A,C,E**).

Unlike menadione treatment, no increases in the proportion of Dye^+^ or CellROX^hi^ cells were detected with H_2_O_2_ treatment in PBS, even at bactericidal concentrations (**Figure 2** and Supplementary Figure S2B). H_2_O_2_ exposures did not change SSC, FSC, or fluorescent profiles of either bacterial species, which suggests minimal contributions by non-specific fluorescence or light scattering (Supplementary Figures S1C,D). CellROX Green is highly sensitive to hydroxyl radicals arising from Fe^2+^/H_2_O_2_-mediated Fenton chemistry, as demonstrated with hydroxyl radicals generated in reactions of ferrous perchlorate (II) with H_2_O_2_ (Invitrogen, 2010, 2012, 2016). So our results suggest that H_2_O_2_-derived hydroxyl radicals and other H_2_O_2_-derived ROS do not reach levels detectable by CellROX Green in H_2_O_2_-treated *E. coli* or *M. smegmatis*. This observation stands in contrast to previous observations in human cells treated with H_2_O_2_ (Segard et al., 2013). However, the fluorescence attributed to CellROX dye in the studies of Segard et al. (2013) is possibly due to autofluorescence or morphologic changes in FSC/SSC caused by extreme protein aggregation in these studies. Changes in autofluorescence and morphology, and not dye activation, similarly accounted for increased fluorescence observed in antibiotic-treated *E. coli* (Renggli et al., 2013; Paulander et al., 2014). Our findings with superoxide-generating menadione and hydroxyl radical-producing H_2_O_2_ support the conclusion that bacterial exposure to even high levels of H_2_O_2_ does not produce hydroxyl radicals at a level detectable by activation of CellROX Green and the conclusion by Choi et al. (2015) that CellROX Green is a sensitive detector of O_2_^•–^ in bacteria.

### Basal Superoxide Levels Depend upon the Bacterial Metabolic State

**FIGURE 3:**
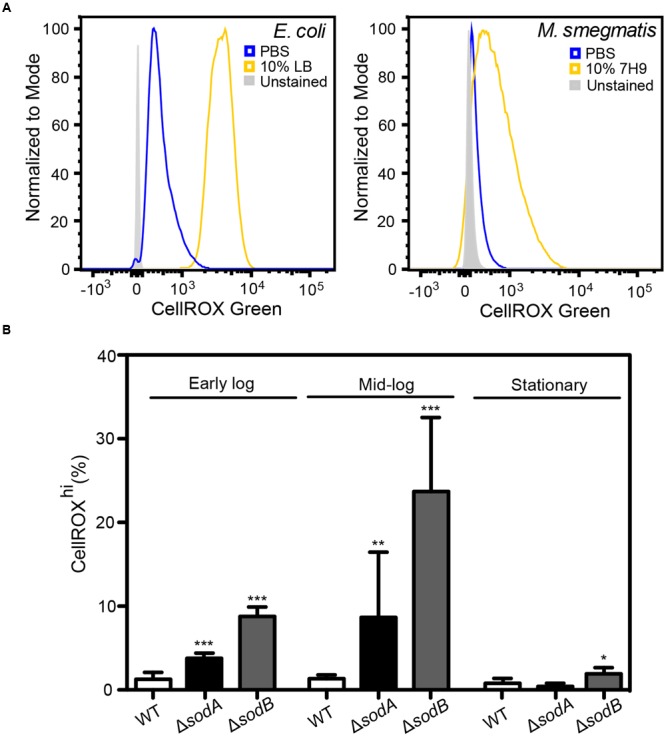
**Basal O_2_^•–^ production in actively dividing *E. coli* and *M. smegmatis.* (A)** Histograms of DNA^+^ *E. coli* and *M. smegmatis* demonstrating increased CellROX Green fluorescence in live bacteria incubated in 10% (v/v) media (LB or 7H9) in comparison to bacteria incubated in PBS. **(B)** Growth stage dependent elevated endogenous O_2_^•–^ in superoxide dismutase (SOD) knockouts (*ΔsodA* and *ΔsodB*). Early log: OD_600_ = 0.2, Mid-log: OD_600_ = 0.6, Stationary: OD_600_ > 2. One-way ANOVA with Dunn’s multiple comparisons versus WT (*E. coli* K-12 BW25113), Bars represent mean ± SEM, *N* = 3, ^∗^*P* < 0.05, ^∗∗^*P* < 0.01, ^∗∗∗^*P* < 0.001.

Here, we tested the ability of CellROX Green to detect changes in O_2_^•–^ levels in cells in different states of respiration and growth. A major source of O_2_^•–^ in respiring cells involves reduction of O_2_ by electrons leaking from the bacterial respiratory chain from NADH dehydrogenase and cytochrome b and at the ubiquinone sites. In *E. coli*, this respiration-dependent O_2_^•–^ production varies according to nutrient levels, at least at early times following changes in nutrient sources and levels (Gonzalez-Flecha and Demple, 1995; Turrens, 2003; Korshunov and Imlay, 2006; Brynildsen et al., 2013; Dwyer et al., 2014; Adolfsen and Brynildsen, 2015; Lobritz et al., 2015). To assess the effects of active respiration on basal O_2_^•–^ levels, we performed CellROX Green staining after transferring actively dividing *E. coli* and *M. smegmatis* into either 10% LB or 7H9 media, respectively, in PBS or into 100% PBS, both after washing twice in their respective media. After 30 min in the absence of LB or 7H9, in which cells have reduced respiration due to acute nutrient deprivation, both *E. coli* and *M. smegmatis* had very low levels of activated CellROX Green fluorescence compared to the background signal from unstained cells (**Figures 3A,B**). However, >95% of both cell types in 10% media/90% PBS showed evidence of dye uptake and a uniform several order-of-magnitude increase in the CellROX^hi^ fluorscence (**Figures 3A,B**). CellROX Green activation observed in cells in the presence of LB or 7H9 media is likely attributable to higher basal metabolism in the actively dividing cells, which leads to respiration-derived O_2_^•–^ (Gonzalez-Flecha and Demple, 1995; Turrens, 2003; Korshunov and Imlay, 2006; Brynildsen et al., 2013; Dwyer et al., 2014; Adolfsen and Brynildsen, 2015; Lobritz et al., 2015).

To verify that metabolically derived O_2_^•–^ is the predominant ROS species reacting with CellROX Green in these actively respiring cells, we quantified dye activation in *E. coli* knockout strains lacking SODs *sodA* and *sodB* and grown in LB to different states of cell density (early log, late log, and stationary phases). Cytosolic O_2_^•–^ is rapidly converted to H_2_O_2_ by the SODs at diffusion-limited rates (k_cat_/K_M_ ∼7 × 10^9^ M^-1^ s^-1^) (Berg et al., 2002). As shown in **Figure 3B**, there is a significant increase in CellROX^hi^ cells among the strains lacking SOD compared to wild-type *E. coli*. Further, by accounting for differences in cell numbers at early, mid-log and stationary cultures by adjusting for turbidity pre-treatment and events collected post-data acquisition (**Figure 3B**), we recapitulated previous observations that metabolic O_2_^•–^ generation correlates with growth stage-dependent cellular metabolic activity rather than overall culture viability (Yamashoji et al., 2001). These results provide further support for the conclusion that CellROX Green activation in bacteria is predominantly due to reactions with O_2_^•–^.

### Antibiotic-Induced Superoxide Production

**FIGURE 4:**
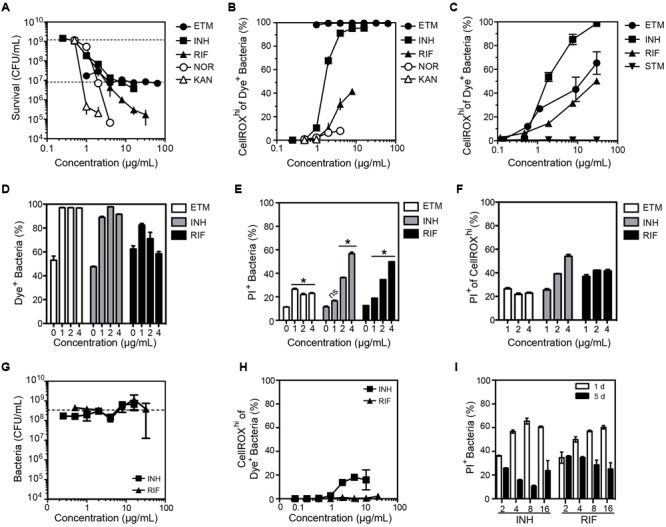
**Changes in the O_2_^•–^ levels in log-growing *M. smegmatis* when exposed to antibiotics. (A)** Bacterial survival after a 24 h exposure to ethambutol (ETM), isoniazid (INH), rifampicin (RIF), norfloxacin (NOR), kanamycin (KAN). Survival for the lowest dose of streptomycin (STM) was below level of detection (1000 CFU/mL) and so STM data is not shown in the graph. Data represent mean ± SEM for *N* = 3. **(B)** Dose-response curves for the proportion of bacteria producing ROS after a 24 h exposure to the indicated antibiotics. Mean ± SEM for *N* = 3. **(C)** Dose-response curves for bacteria producing ROS after a 3 h exposure to antibiotics. Mean ± SEM for *N* = 3. **(D)** Percentage of DAPI^+^ cells with CellROX Green Dye fluorescence above background (Dye^+^). Percentage of bacterial cells **(E)** and CellROX^hi^ cells **(F)** stained with propidium iodide (PI^+^; cell membrane integrity index) as a function of antibiotic dose after 24 h of exposure. Mean ± SEM for *N* = 3. One-way ANOVA with Dunn’s multiple comparisons versus untreated, ^∗^*P* < 0.05. **(G)** Selection of antibiotic-resistant clones of *M. smegmatis* by growth in the presence of isoniazid (INH) and rifampicin (RIF) for 5 days. The sensitivity of the resulting bacteria to INH and RIF was assessed by a CFU assay, with data representing mean ± SEM for *N* = 3. The dashed line shows the CFU for untreated bacteria passaged for 5 days (2 × 10^8^ CFU/mL). **(H)** Proportion of *M. smegmatis* producing CellROX Green-detectable ROS after 5 days of exposure to antibiotics at the indicated concentrations; data represent mean ± SEM for *N* = 3. **(I)** Proportion of *M. smegmatis* with PI staining after a 1 or 5 days exposure to antibiotics at indicated concentrations (μg/mL). Mean ± SEM for *N* = 3.

There is emerging and intriguing evidence for multiple roles for ROS in the bacterial response to antibiotic exposure (Nguyen et al., 2011; Grant et al., 2012; Dwyer et al., 2015). However, methods using ROS-reactive dyes to identify and quantify ROS in antibiotic-treated bacteria are prone to artifacts in the absence of rigorous controls. For example, drug-induced changes in cell morphology can increase background fluorescence instead of activating the dye (Kohanski et al., 2007; Renggli et al., 2013; Paulander et al., 2014), the use of ROS-reactive dyes in batch culture methods does not inform about ROS inside cells (Albesa et al., 2004), and there is disagreement about the ability of ROS-reactive dyes to gain entrance into bacteria such as *Pseudomonas aeruginosa* and *M. smegmatis* (Nguyen et al., 2011; Grant et al., 2012). In all of these cases, rigorous flow cytometric gating to control for dye uptake, cell morphology and background fluorescence could minimize these problems. Here, we illustrate this idea using CellROX Green to assess the relationship between ROS production and cytotoxicity in *M. smegmatis* treated with mechanistically distinct antibiotics known to be effective against mycobacteria: ethambutol (cell wall synthesis inhibitor), streptomycin (binds to 30S ribosome subunit), rifampicin (binds to DNA-dependent RNA polymerase), isoniazid (inhibits mycolic acid synthesis), kanamycin (binds to 30S ribosome subunit), and norfloxacin (topoisomerase inhibitor). *M. smegmatis* cytotoxicity dose-response curves for these antibiotics are shown in **Figure 4A**.

We first assessed the effects of antibiotic exposures on FSC, SSC and background fluorescence in *M. smegmatis*. As shown in Supplementary Figure S3, while there were no consistent changes in SSC or increased background fluorescence (in absence of dye), there were time-, dose- and antibiotic-dependent shifts to lower SSC in treated bacteria. Given the recognized link between SSC shift and bacterial cell cycle progression (Walberg et al., 1997a,b), elongation (Renggli et al., 2013), and the uniquely asymmetric cell division of rod-shaped mycobacteria (Kieser and Rubin, 2014), these results are consistent with a delayed cell elongation in *M. smegmatis* populations hours after the initial drug exposure.

After accounting for morphological changes in and background fluorescence, we next assessed antibiotic-induced O_2_^•–^ generation in *M. smegmatis* using CellROX Green. As shown in **Figures 4B,C**, antibiotics did not consistently cause O_2_^•–^ generation in *M. smegmatis*. While ethambutol, rifampicin, and isoniazid increased CellROX^hi^ levels after 24 h of treatment (**Figure 4B**) and as early as 3 h after treatment (**Figure 4C**), norfloxacin and kanamycin treatments produced minimal or no dye-detectable ROS in response to 24 h treatment (**Figure 4B**). For streptomycin, no dye-detectable ROS was observed after 3 h of exposure (**Figure 4C**), while too many cells were killed at 24 h for accurate measurements.

Quantitative relationships between CellROX activation and cytotoxicity caused by the antibiotics were observed to be inconsistent in three studies. First, treatment with ethambutol produced dose-dependent killing while >95% of the cells produced dye-detectable ROS across all tested doses (**Figures 4A,B**). We then assessed antibiotic-induced toxicity by co-staining with propidium iodide (PI), a dye that is excluded from viable cells but enters cells with damaged membranes, and by survival as colony-forming units (CFUs). Based on flow cytometry contour plots, such as those in Supplementary Figures S4A–C, uptake of PI and CellROX varied among antibiotic exposures. However, similar proportions of cells were stained with PI in both the total population (**Figure 4E**) and the CellROX^hi^ population (**Figure 4F**) at 24 h post-exposure. In a third set of studies, the inconsistency of ROS formation and cell toxicity was more pronounced in CFU cell death assays for both *E. coli* and *M. smegmatis* (Supplementary Figure S4D). Together, these data suggest that the mechanisms by which antibiotics cause increased O_2_^•–^ production are unique to each antibiotic, and elevated steady-state O_2_^•–^ levels alone are not causative or predictive of cell death.

Finally, we assessed the formation of O_2_^•–^ in *M. smegmatis* clones induced to be resistant to isoniazid and rifampicin, two antibiotics that caused significant O_2_^•–^ production (**Figures 4B,C**). Antibiotic-tolerant bacteria were generated by culturing *M. smegmatis* in varying concentrations of antibiotics for 5 days. Despite several logs of killing by 20–30 μg/mL isoniazid and rifampicin at 24 h (**Figure 4A**), 5 days of growth in the presence of the antibiotics led to a drug-resistant population (**Figure 4E**). Compared to *M. smegmatis* treated for 24 h, the antibiotic-tolerant bacteria displayed greatly reduced O_2_^•–^ production following antibiotic exposures (**Figure 4F**). Furthermore, there were fewer PI^+^ bacteria (i.e., bacteria with damaged membranes) and no positive correlation between PI staining and antibiotic concentration at 5 days was observed, in contrast with the same treatment after 24 h (**Figures 4E,G**). The mechanisms by which bacteria became resistant to the antibiotics was not established.

### Nutrient Deprivation Induces Superoxide Production and Antibiotic Tolerance in Mycobacteria

**FIGURE 5:**
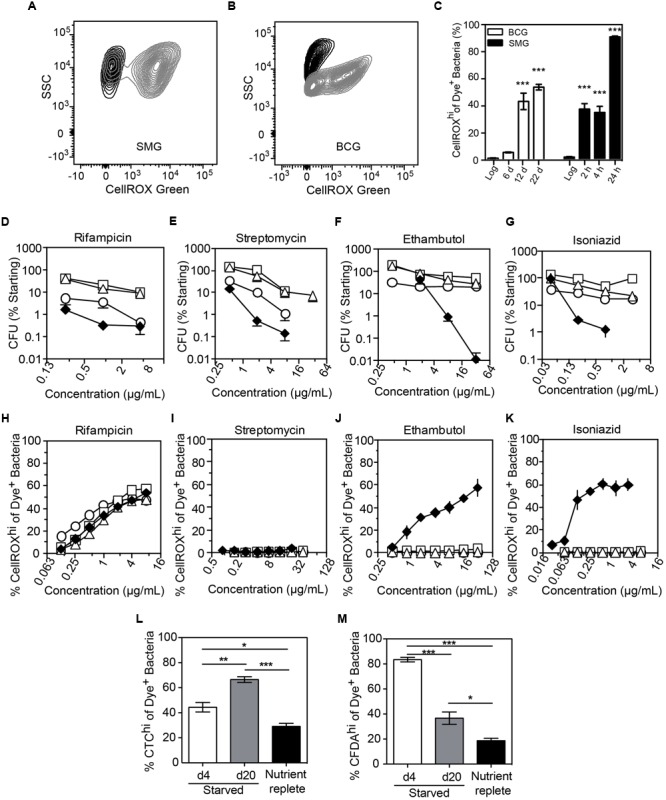
**Endogenous and antibiotic-induced O_2_^•–^ in starved, non-replicating *M. bovis* BCG and *M. smegmatis*.** Representative contour plots of log-growing (black) and starved (gray) DNA^+^ bacteria in cultures of *M. smegmatis* (**A**, 24 h starvation) and *M. bovis* BCG (**B**, 20 days starvation). **(C)** The starvation-induced O_2_^•–^ production in *M. smegmatis* (SMG) and *M. bovis* BCG populations were quantified and presented. Data represent mean ± SEM for *N* = 3, with one-way ANOVA with Dunn’s multiple comparisons versus untreated, ^∗∗∗^*P* < 0.001. *M. bovis* BCG survival after 48 h of treatment with rifampicin **(D)**, ethambutol **(E)**, streptomycin **(F)** and isoniazid **(G)** during log growth (♦) and nutrient deprivation for 4 days (○), 10 days (□), or 20 days (Δ); data represent mean ± SEM for *N* ≥ 3. (**H–K**) O_2_^•–^ production above basal levels in log (♦) and starved (4 days, ○; 10 days, □; 20 days, Δ) *M. bovis* BCG exposed to antibiotics for 48 h; data represent mean ± SEM for *N* ≥ 3. Respiratory (**L**, CTC dye) and esterase (**M**, CFDA dye) activities in non-replicating (starved for 4 and 20 days in PBS + 0.05% Tyloxapol) and replicating (10 days in nutrient-replete 7H9 media) *M. bovis* BCG. Data represent mean ± SEM for *N* ≥ 3. ^∗^*P* < 0.05, ^∗∗^*P* < 0.01, ^∗∗∗^*P* < 0.001 by unpaired, two-tailed, *t*-test as indicated.

We next assessed O_2_^•–^ generation in mycobacteria in the well-established non-replicating and antibiotic-tolerant (i.e., persistent) physiological state caused by nutrient deprivation (Xie et al., 2005; Gengenbacher et al., 2010). Interestingly, starved bacteria consistently produced significantly higher levels of endogenous O_2_^•–^ than log-growing, nutrient-replete cells (**Figures 5A–C**). This was not a quirk of *M. smegmatis* as we observed the same phenomenon in slow-growing *M. bovis* BCG when starved up to 22 days (**Figures 5A–C**). Given this starvation-induced increase in basal O_2_^•–^ production and the observation that increased ROS levels render some bacteria susceptible to killing by specific antibiotics (Brynildsen et al., 2013; Dwyer et al., 2014; Adolfsen and Brynildsen, 2015; Belenky et al., 2015), we quantified antibiotic sensitivity of log-growing or starved (4, 10, 20 days) BCG with the front-line TB antibiotics isoniazid, rifampicin, ethambutol, or streptomycin. As shown in **Figures 5D–G**, starved mycobacteria were tolerant to all four antibiotics compared to log-growing bacilli in spite of elevated basal O_2_^•–^ production. This idiosyncratic behavior of starvation-induced persisters led us to investigate the effect of antibiotic exposure on O_2_^•–^ levels in these cells. After accounting for elevated basal O_2_^•–^ caused by starvation, we found that generation of O_2_^•–^ in antibiotic-stressed, starved BCG was idiosyncratic to the antibiotic. While higher than basal levels of O_2_^•–^ were observed in log-growing BCG for isoniazid and ethambutol (**Figures 5J,K**), as observed for *M. smegmatis* (**Figures 4B,C**), elevated O_2_^•–^ generation above the basal level was not observed in starved BCG treated with streptomycin, isoniazid, or ethambutol (**Figures 5I–K**), which agrees with other studies on antibiotic-related ROS in persisters (Nguyen et al., 2011; Grant et al., 2012).

A possible explanation for the lack of antibiotic-related endogenous O_2_^•–^ induction in starvation-induced persisters is metabolic quiescence. To test for metabolic activity in starved cells, we performed staining of starved BCG with CTC, CFDA, and DAPI. Enhanced respiratory activity was observed in a sub-population of cells, as evident in the increase of the CTC^hi^ sub-population during starvation and the reversal of CTC activation by incubation in nutrient-replete medium (**Figure 5L** and Supplementary Figure S5A). Starvation also increased cellular esterase activity as shown by a CFDA^hi^ sub-population (**Figure 5M** and Supplementary Figure S4A), which again is reversed by transfer to nutrient-replete medium. Together, these observations eliminate metabolic stasis as the cause of the muted ROS production in the response of starved BCG antibiotic exposure.

Another possible explanation for reduced O_2_^•–^ generation in antibiotic-exposed non-replicating mycobacteria is the active exclusion of drugs through efflux pumps. Indeed, drug efflux pumps are often up-regulated in drug-tolerant cells (Sarathy et al., 2013; Maisonneuve and Gerdes, 2014). However, we observed that rifampicin treatment induced O_2_^•–^ generation in a dose-dependent manner in both log and starved BCG (**Figure 5H**), in spite of starvation-induced up-regulation of transcription of a known rifampicin efflux pump, BCG_1316c (*M. tuberculosis* Rv1258c homolog) (Adams et al., 2011) (Supplementary Figure S5B). Even with enhanced O_2_^•–^ production above an already high basal level, rifampicin treatment did not result in cell death (**Figure 5D**), indicating that starved BCG is refractory to O_2_^•–^-mediated killing, unlike log-growing *E. coli* (Brynildsen et al., 2013; Dwyer et al., 2014; Adolfsen and Brynildsen, 2015; Belenky et al., 2015). These findings demonstrate that antibiotic-induced O_2_^•–^ production and associated killing is both antibiotic- and cell state-dependent.

## Discussion

The use of fluorescent probes combined with flow cytometry is a powerful approach to investigate biological processes within single cells as well as in cell populations. We developed a four-step gating strategy to minimize false positive signals and artifacts by accounting for DNA content, changes in cell morphology, dye uptake and retention, and target specific dye activation in bacterial cells. This approach is generally applicable to intracellular metabolic probes, as illustrated here with CellROX Green, CTC and CFDA-AM. With specific application to CellROX Green, the flow cytometry method provides high-dimensional quantitative measurement of light scatter and fluorescence emission by this O_2_^•–^-activated fluorescent probe, thus allowing measurement of O_2_^•–^ in 1000s of individual cells within a sample in addition to the population abundance.

The four-step gating scheme presented here makes use of several well-accepted gating strategies for mammalian cells and systematically applies them to bacteria at each of the four steps. The basis for *Step 1* is the selection of the cell population of interest based on scatter properties, such as when selecting for lymphocytes versus monocytes or granulocytes. Although commonly used in environmental microbiology, scatter properties of bacteria have not been considered in ROS probe-based studies. DNA content staining, as in *Step 2*, although not absolutely necessary, strengthens the precision and accuracy of the data by distinguishing bacteria from particles of similar sizes. It also provides an additional parameter for analysis along with FSC and SSC, which together can distinguish different bacteria or bacteria in different cell states (Muller and Nebe-von-Caron, 2010). Finally, DNA content gives information about life cycle stages within the population that may provide insight into stress response mechanisms.

While large aggregates and dramatic filamentation would be excluded in our plots due to the flow cytometer settings for FSC (only particles <10 um are collected during analysis), the gating scheme does not necessarily ignore elongated cells and instead accounts for their elongation. By taking into account increases in fluorescence observed in cells with higher SSC or FSC (done by two-dimensional gating such as SSC versus fluorescence), cells with increased SSC or FSC can be selected for analysis if they pass the subsequent gates for increases in green fluorescence to be considered Dye^+^ and then CellROX^hi^. This gating procedure normalizes for changes in SSC by not using flat cutoffs as in histograms (e.g., cut-off at fluorescence of 2 × 10^3^). If one does see two distinct populations of cells, separate size gates (FSC, SSC) or DNA content gates can be made for each population followed by consistent gating for green fluorescence. The changes in background green fluorescence are not the same magnitude as activated CellROX Green dye, so generation of ROS in cells with high background staining should be distinguishable using unstained and stained treated cells.

Together, *Steps 3* and *4* of the gating scheme select for probe fluorescence inside cells, which validates the location of the probe reaction with ROS. *Step 3* selects all cells with fluorescence above no-dye controls. This basal fluorescence must be considered a conservative, baseline signal that accounts for detection of unreacted, reacted, or degraded dye in the cell or, less likely, adhering to the cell surface, as well as fluorescence from intracellular components. Considering that culture conditions, bacterial species, and stress can affect dye permeability in some bacteria (Walberg et al., 1999), an assessment of dye content in each cell becomes important, whenever possible. The point of gating *Step 3* is a conservative one: to minimize noise and artifacts by including in the analysis only cells that appear to have taken up a dye molecule. By setting the selection threshold for *Step 4* above these basal levels, *Step 4* selects cells with a high probability of containing an ROS-activated dye molecule. It is important to use the Dye^+^ gate as the denominator for determining positive ROS signals as different stress treatments change the basal fluorescence.

There are several advantages to this a four-step gating flow cytometry method. ROS in bacterial populations have been evaluated with fluorescent dyes using plate readers or by measuring mean fluorescence intensity (MFI) in total cell populations collected using a flow cytometer (Kohanski et al., 2007; Nguyen et al., 2011; Dwyer et al., 2012, 2014). However, the problem with these approaches is that, depending upon the gating of the flow cytometer data, the use of controls, and rigorous characterization of the dye sensitivity and specificity, these fluorescence methods can yield false positives due to background fluorescence from the dye or the treatment, as reported previously and observed in the present studies (Renggli et al., 2013; Paulander et al., 2014). Plate reader data also suffers from artifacts of extracellular fluorescence. Moreover, mean or median changes in fluorescence in total populations may reflect a small proportion of cells producing high levels of ROS or a large proportion generating low to moderate amounts of ROS. Given evidence for the intracellular ROS concentration as a determinant of cellular responses to specific antibiotics (Brynildsen et al., 2013), the ability to both identify ROS and measure ROS abundance at the single cell level is vital to understanding mechanisms of cellular responses. The strategy presented here for detection of ROS with a flow cytometer combined with rigorous gating of populations allows for determination of relative abundance of ROS generation within single cells. One of the advantages of using CellROX Green activation as a O_2_^•–^ index in bacteria involves retention of activated dye in cells by binding to DNA, which prevents loss of activated probe through efflux pumps or leakage through damaged cell membranes.

In terms of the chemical identify of the ROS species detected by CellROX Green, we conclude that this dye selectively detects O_2_^•–^ in bacterial populations under a variety of environmental conditions and exposures. Our results with menadione exposure and SOD mutant strains are consistent with published studies pointing to CellROX Green specificity for O_2_^•–^ in bacterial cells (Choi et al., 2015). We cannot rule out dye activation by RNS such as peroxynitrite (ONOO^-^), the reaction product of NO and O_2_^•–^, which decays to generate nitrogen dioxide radical (^∙^NO_2_) and hydroxyl radical (Dedon and Tannenbaum, 2004). However, it is unlikely that, even with high O_2_^•–^ levels, there would be significant ONOO^-^ formation in bacteria under the exposure conditions used here. The relative insignificance of hydroxyl radicals generated from ONOO^-^ is further emphasized by the fact that even high, supra-lethal concentrations of H_2_O_2_, which is well-established to react with reducing metals to generate cytotoxic hydroxyl radicals or equivalently reactive ferryl radicals (Imlay et al., 1988; Imlay, 2015), did not produce detectable activation of CellROX Green. This points to either a relative insensitivity of the dye to hydroxyl radicals *in vivo* or to production of significantly lower levels of hydroxyl radicals relative to O_2_^•–^ in the cells. H_2_O_2_ can also lead to indirect ROS and RNS production in cells by activating redox-sensitive transcription factors, such as OxyR and PerR in bacteria, to remodel cell metabolism and energetics, with resulting increases in O_2_^•–^ and other dye-reactive radical species (Marinho et al., 2014). Again, the lack of H_2_O_2_-induced CellROX Green activation rules out such a mechanism. H_2_O_2_-induced metabolic remodeling may explain the activation of CellROX Green in H_2_O_2_-exposed human cells (Segard et al., 2013). However, our studies reveal a lack of dye-detectable ROS species in bacteria exposed to supra-lethal levels of H_2_O_2_. Clearly the interpretation of studies using H_2_O_2_ as a calibrant in dye-based studies of ROS generation should be approached with caution.

The results of our studies with *E. coli* and *M. smegmatis* reveal several types of heterogeneity and idiosyncrasy in the bacterial ROS responses to chemical stimuli, which warrant customization of the dye-based ROS method and caution in interpreting the results. For example, both types of bacteria showed different responses to menadione as a population (visualized in fluorescence histograms – **Figures 2B,D**). With increasing menadione exposures, a dose-dependent increase in size of the CellROX^hi^ *M. smegmatis* sub-population was observed. However, there was no change in the population distribution as demonstrated by the absence of any change in the mean, median or mode fluorescence intensity (**Figure 2B**). In contrast, two distinct populations emerged during menadione stress for *E. coli*. This change in population distribution was most evident at high menadione concentrations when a significant increase in the mean, median and mode fluorescence intensity between low and high menadione exposures was observed (**Figure 2D**). While we do not have a mechanistic explanation for this behavior, we note that it is a consistent and reproducible shift in the size of the CellROX^hi^ subpopulation in response to menadione indicating O_2_^•–^ stress. It cannot be ruled out, however, that in longer exposures to menadione, further fluorescence shifts in the population of both *E. coli* and *M. smegmatis* would occur. However, under current conditions, the two bacteria both produce CellROX Green-detected ROS, but with different staining patterns. These differences between *E. coli* and *M. smegmatis* suggest caution in extrapolating ROS results across bacterial species and genera. In some cases, it is not even possible to compare across different types of bacteria to due dye accessibility problems. We shared the same problem experienced by Grant et al. (2012) that HPF could not be used to detect ROS in *M. smegmatis* even though it works with *E. coli*.

This same caution in interpreting the results of ROS dye signals and extrapolating results across species must be applied to studies of antibiotic pharmacodynamics. While fluorescent probes have been used to explore ROS generation caused by antibiotic exposure (Kohanski et al., 2007; Nguyen et al., 2011; Dwyer et al., 2012, 2014; Grant et al., 2012), there is no unifying mechanistic model to explain why ROS are generated with specific antibiotics in some bacterial species but not others, and, more importantly, which type of ROS is generated. Our findings with CellROX Green in *M. smegmatis* and *E. coli* under the variety of conditions that cause increases in dye-detectable ROS lead us to conclude that O_2_^•–^ generation (1) is not a universal feature of antibiotic stress, (2) does not distinguish bactericidal from bacteriostatic antibiotics, and (3) is not generally predictive of cell death (**Figure 4I**). Rather, our data suggest that ROS production is dependent on the physiological state of the cell, specifically on changes in metabolism. Previous studies of ROS metabolism in antibiotic tolerant bacteria revealed clear differences in the drug-host interaction (Nguyen et al., 2011; Maisonneuve and Gerdes, 2014). Similarly, our observation of O_2_^•–^ production during rifampicin treatment may be related to enzyme induction and metabolism, as the antibiotic is a potent inducer of human cytochrome P450 (CYP) enzymes, most notably CYP3A4 and CYP2C subfamily (Madan et al., 2003). This suggests that rifampicin may also induce CYPs in mycobacteria (Niemi et al., 2003). Since CYP activity is well-known to generate high levels of O_2_^•–^ (Denisov et al., 2005; Munro et al., 2013), our observation of dose-dependent CellROX Green activation in both log-growing and starved bacilli following rifampicin exposure (**Figures 4B, 6B**) is consistent with previous studies showing transcriptional up-regulation of different CYPs in mycobacteria following exposure to a variety of antibiotics (Boshoff et al., 2004). Indeed, recently released microarray datasets for transcriptional response to antibacterial agents in the closely related human pathogen, *M. tuberculosis*, shows that several CYPs, including *cyp51* (Rv0764c), *cyp124* (Rv2266), *cyp135A1* (Rv0327c) and *cyp139* (Rv1666c), are upregulated in response to rifampicin (NCBI Gene Ontology Omnibus Accession: GSE71200).

The presence of elevated basal ROS levels in starved mycobacteria might suggest a vulnerability in light of evidence that antibiotic-induced “ROS” potentiate existing ROS to promote bacterial cell death (Brynildsen et al., 2013; Dwyer et al., 2014; Adolfsen and Brynildsen, 2015; Belenky et al., 2015). However, our observations in mycobacteria do not support this as a universal model. For instance, the non-replicating, antibiotic-resistant mycobacteria generated through nutrient deprivation have elevated O_2_^•–^ (**Figure 5D**), yet are significantly resistant to four front-line tuberculosis antibiotics (**Figures 5D–G**). While endogenous ROS production has been correlated with antibiotic killing in some cases (Brynildsen et al., 2013; Dwyer et al., 2015), increases in intracellular O_2_^•–^ levels in starved mycobacteria failed to potentiate the activities of several antibiotics. This observation also raises concerns about the conclusion that the O_2_^•–^-generating tuberculosis antibiotic clofazimine, which also binds tightly to DNA and RNA, facilitates INH-induced killing of persistent *M. smegmatis* by virtue of O_2_^•–^ production (Grant et al., 2012). It is possible that clofazimine’s cytotoxic activity involves tight binding of its reduced, free radical form to DNA and RNA, as demonstrated for the parent compound (Morrison and Marley, 1976), to create covalent adducts. In any event, our results demonstrate that O_2_^•–^ is unlikely to be the cause of toxicity in persistent or drug-resistant non-replicating mycobacteria.

Finally, we address the impact of potential heterogeneity in what appears to be toxicant-induced changes in uptake of dye molecules into individual cells. This issue becomes apparent, for example, in the increased proportion of cells appearing in the Dye^+^ gate following menadione treatment (**Figure 2E**), which correlates with the proportion of cells with a fluorescence signal greater than untreated cells (CellROX^hi^; **Figures 2A,C**). What appears to be a toxicant-induced increase in the number of cells taking up dye raises the concerns that an increased CellROX^hi^ signal could occur in spite of constant toxicant-independent ROS levels (more dye molecules to activate) or as a result of a large influx of unactivated, weakly fluorescent parent dye molecules. It is important to point out that the flow cytometry method is limited in its ability to precisely quantify the contribution of toxicant-induced increases in dye uptake of individual cells, such as increased permeability to CellROX Green or reduced efflux of the dye. However, the nature of the gating strategy, the cell permeability and spectroscopic properties of the fluorescent dye, and our observations of membrane permeability changes using PI argue that stress-induced increases in CellROX Green fluorescence are truly due to increases in O_2_^•–^ levels in the cells. First, the Dye^+^ gate precedes CellROX^hi^ gating, so the latter is a subset of the former and, in a rising tide argument, increases in the Dye^+^ population will naturally accompany increases in the CellROX^hi^ population. The Dye^+^ gate of increased fluorescence above unstained cells is intended to be a conservative barrier to rule out artifacts, so it is possible that untreated cells taking up smaller amounts of the weakly fluorescent dye will be gated out of the analysis for the sake of accuracy at the cost of sensitivity, while increases in ROS levels in treated cells will raise the fluorescence of these previously missed low-dye cells above the threshold for Dye^+^. Second, our analysis of antibiotic exposure and ROS in **Figure 4** revealed that there is no correlation between antibiotic-induced changes in membrane permeability (PI staining) and the magnitude of the CellROX^hi^ signal: ETM caused the highest CellROX Green fluorescence (**Figures 4B,C**) and the lowest membrane damage (**Figures 4E,F**). Third, CellROX Green is a cell-permeant dye, so its concentration should equilibrate between bulk solvent and cell interior. The spectroscopic properties of CellROX Green imply that very high levels of unactivated dye must accumulate in a cell to produce a signal that rivals the magnitude of ROS-activated dye. Assuming that CellROX Green has spectral properties roughly similar to HPF, then both molar absorptivity and quantum efficiency are greatly increased by ROS activation: >3-fold and >100-fold, respectively (Setsukinai et al., 2003). This implies that an approximately >1000-fold increase in intracellular CellROX Green concentration would be needed to produce the equivalent of a 10-fold increase in ROS-activated CellROX Green fluorescence above the mean (or median or mode) fluorescence of the population. The additional requirement for DNA binding to achieve the enhanced fluorescence by ROS-activated CellROX Green (Invitrogen, 2010, 2012, 2016) further reduces the likelihood that unactivated dye could account for toxicant-induced changes in CellROX^hi^. This is also consistent with the observation by Choi et al. (2015) that permeabilization of the *E. coli* cell membrane/wall by Triton X-100 did not alter CellROX Green fluorescence. Finally, if the increased fluorescence in cells were simply due to increased dye concentration, then there would not be multiple distinct cell populations observed on the flow cytometer (e.g., **Figure 2D**). All of this evidence rules out toxicant- and stress-induced changes in dye uptake as the basis for increased CellROX^hi^ detection of ROS.

Taken together, the results of our studies demonstrate that properly controlled flow cytometry coupled with fluorescent probes provides precise and accurate quantitative analysis of ROS generation and metabolic changes in stressed bacteria. The gating strategy provides for a consistent comparison based on specific parameters across multiple samples in spite of changes in cellular morphology or background fluorescence. Application of this method with CellROX Green and diverse bacteria reveals that superoxide generation arises idiosyncratically due to transient or phenotypic alterations in cell physiology caused by environmental or chemical stresses and is itself not a primary driver of bacterial death.

## Author Contributions

MM, YC, MS, and PD designed experiments. MM, YC, MS, PH, and MC performed experiments. MM, YC, MS, PH, and MC analyzed data. MM, YC, MS, PH, and PD wrote the manuscript. MM, YC, and MS made an equal contribution.

## Conflict of Interest Statement

The authors declare that the research was conducted in the absence of any commercial or financial relationships that could be construed as a potential conflict of interest.
