# Supplementary material for: Production of Superoxide in Bacteria Is Stress- and Cell State-Dependent: A Gating-Optimized Flow Cytometry Method that Minimizes ROS Measurement Artifacts with Fluorescent Dyes
Source: Front Microbiol. 2017 Mar 21;8:459. doi: 10.3389/fmicb.2017.00459 (PMC5359317; doi:10.3389/fmicb.2017.00459)
Supplement: Supplementary file 1 [file Image_1.pdf]

frontiers

Supplementary Material

Production of Superoxide in Bacteria is Stress- and Cell State-dependent: A Gating-optimized Flow Cytometry Method that Minimizes ROS Measurement Artifacts with Fluorescent Dyes

Megan E. McBee, Yok Hian Chionh, Mariam Lucila Sharaf, Peiying Ho, Maggie Wei Ling Cai and Peter C. Dedon*

*** Correspondence:** Corresponding Author: email@uni.edu

Supplementary Figures:

Supplementary Figure S1
Supplementary Figure S2
Supplementary Figure S3
Supplementary Figure S4
Supplementary Figure S5

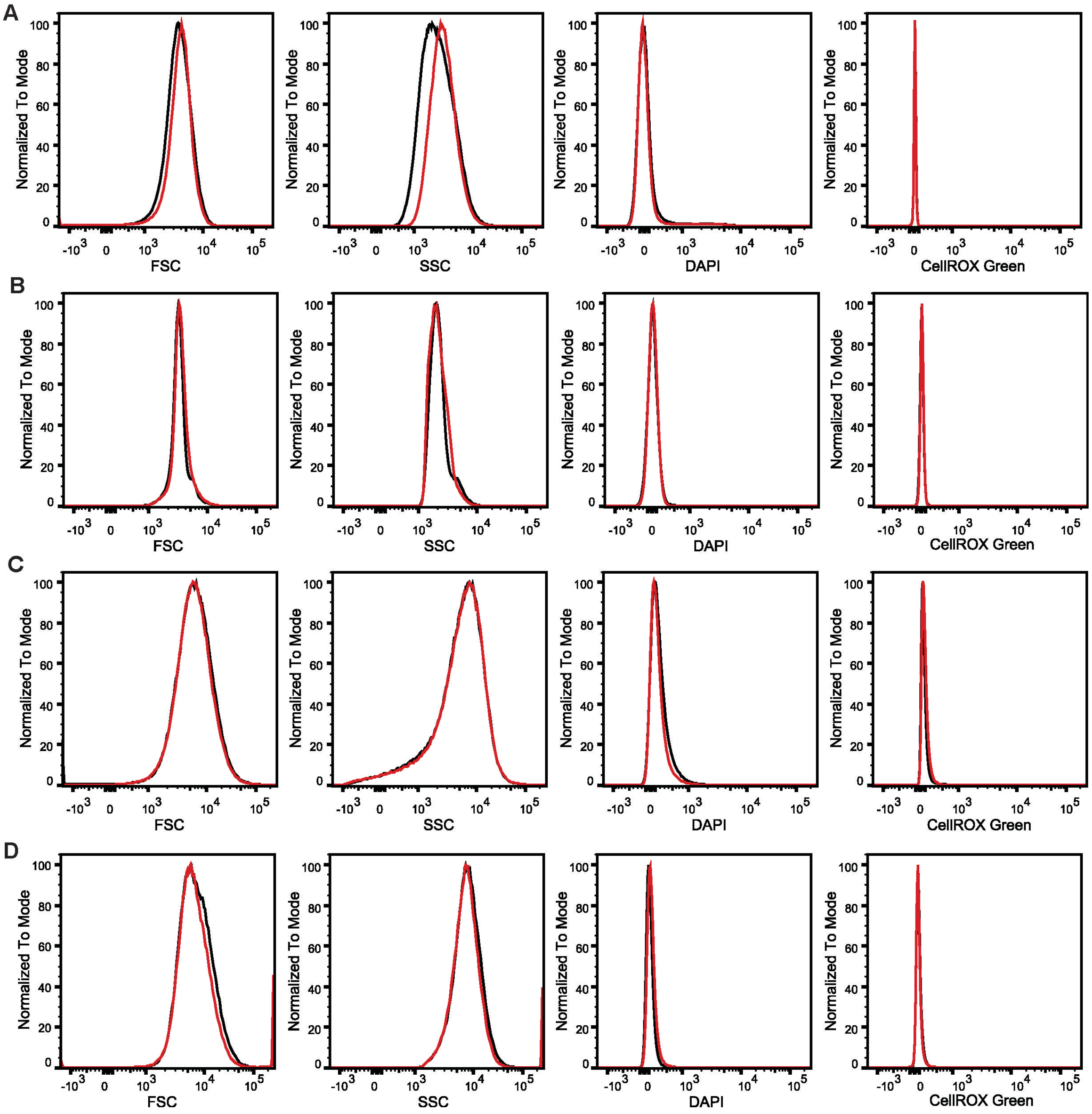

Supplementary Figure 1. Scatter and fluorescence profiles of *M. smegmatis* and *E. coli* are not altered by menadione or H_2O_2 treatment. Forward-scatter (FSC), side-scatter (SSC), CellROX Green and DAPI fluorescence histograms were obtained for (**A**) *M. smegmatis* after 1 h exposure to 100 μM menadione (red) or untreated (black), (**B**) *E. coli* after 1 h exposure to 100 μM menadione (red) or untreated (black) (**C**) *M. smegmatis* after 1 h exposure to 2 mM H_2O_2 (red) or untreated (black) and (**D**) *E. coli* after 1 h exposure to 2 mM H_2O_2 (red) or untreated (black).

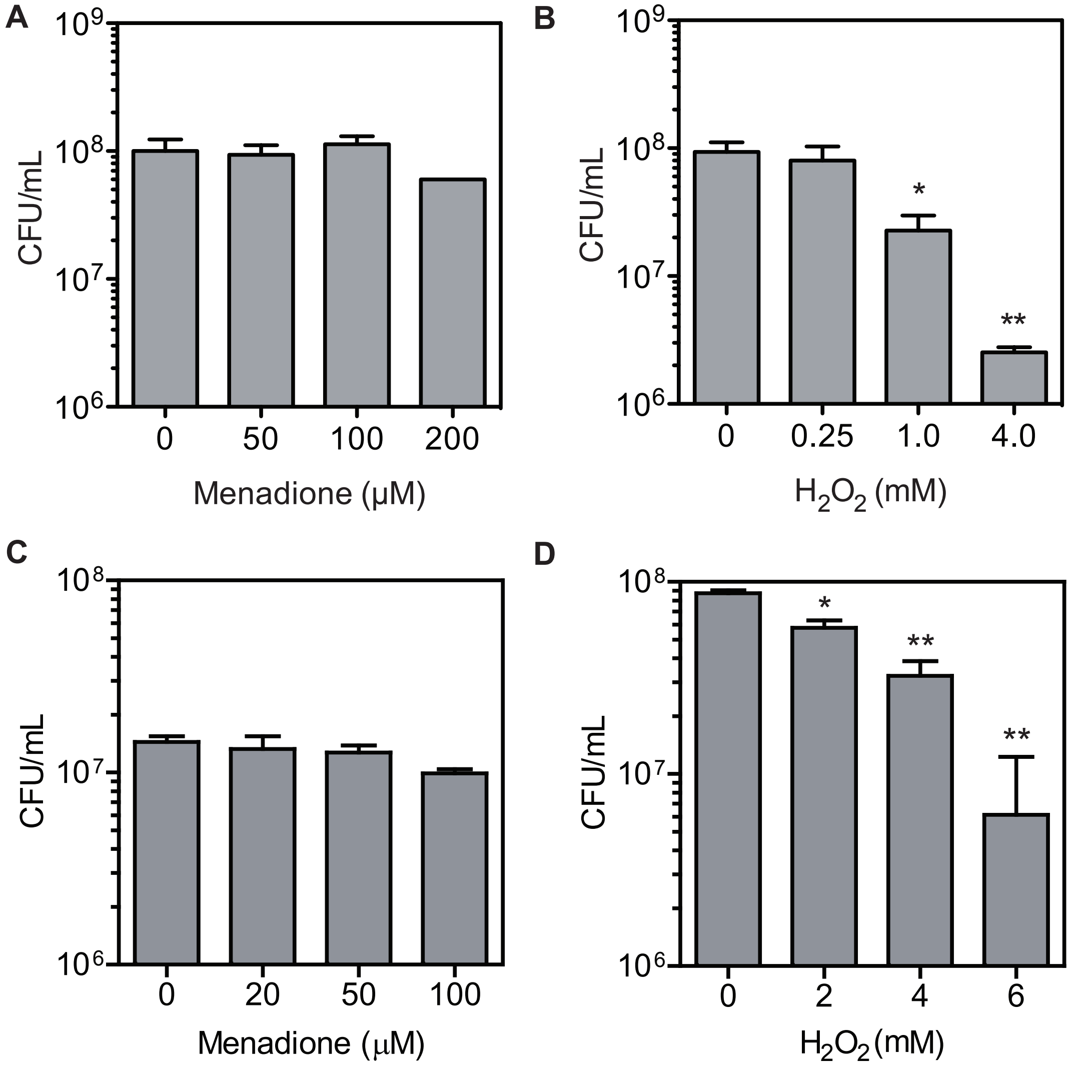

Supplementary Figure S2. Survival of *E. coli* and *M. smegmatis* to menadione and H_2O_2. Survival of *E. coli* BW25113 after 1 h exposure to (A) menadione and (B) H_2O_2 in phosphate-buffered saline (PBS). Survival of *M. smegmatis* after 2 h exposure to menadione (C) and 1 h exposure to H_2O_2 (D) in phosphate-buffered saline (PBS). Mean ± SEM. N > 3. One-way ANOVA with Bonferroni multiple comparisons test. * $P < 0.05$, ** $P<0.01$.

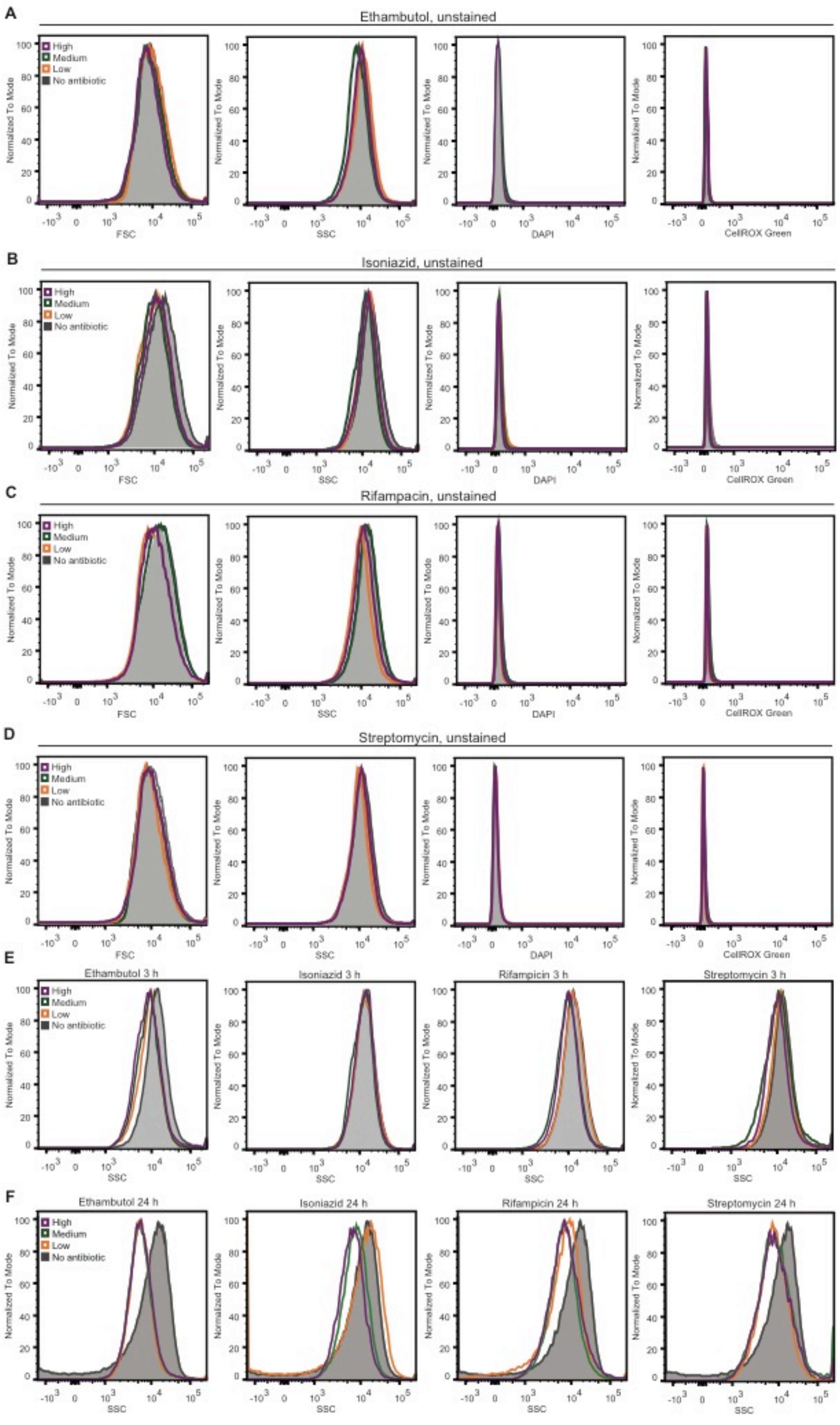
A
Ethambutol, unstained
B
Isoniazid, unstained
C
Rifampacin, unstained
D
Streptomycin, unstained
E
Ethambutol 3 h
Isoniazid 3 h
Rifampicin 3 h
Streptomycin 3 h
F
Ethambutol 24 h
Isoniazid 24 h
Rifampicin 24 h
Streptomycin 24 h
High
Medium
Low
No antibiotic
Normalized To Mode
FSC
SSC
DAPI
CellROX Green

Supplementary Figure S3. Side-scatter shifts in *M. smegmatis* cultures under antibiotic stress. **(A-D)** Forward scatter (FSC), side scatter (SSC), DAPI channel, and CellROX channel histograms of *M. smegmatis* treated with antibiotics at the following concentrations (high, medium, low): ethambutol (18, 2.2, 0.14 μg/mL), isoniazid (15, 3.8, 0.23 μg/mL), rifampicin (15, 3.8, 0.23 μg/mL), and streptomycin (15, 3.8, 0.23 μg/mL). Gray shaded histogram represents untreated cells. **(E)** Side-scatter (SSC) histograms of *M. smegmatis* after 3 h exposure to antibiotics at the same concentrations as in **A-D.** Gray shaded histogram represents untreated cells. **(F)** SSC histograms of *M. smegmatis* after 24 h exposure to antibiotics at the following concentrations (high, medium, low): ethambutol (32, 8, 2 μg/mL), isoniazid (8, 2, 0.5 μg/mL), rifampicin (16, 4, 1 μg/mL), and streptomycin (16, 4, 1 μg/mL). Gray shaded histogram represents untreated cells.

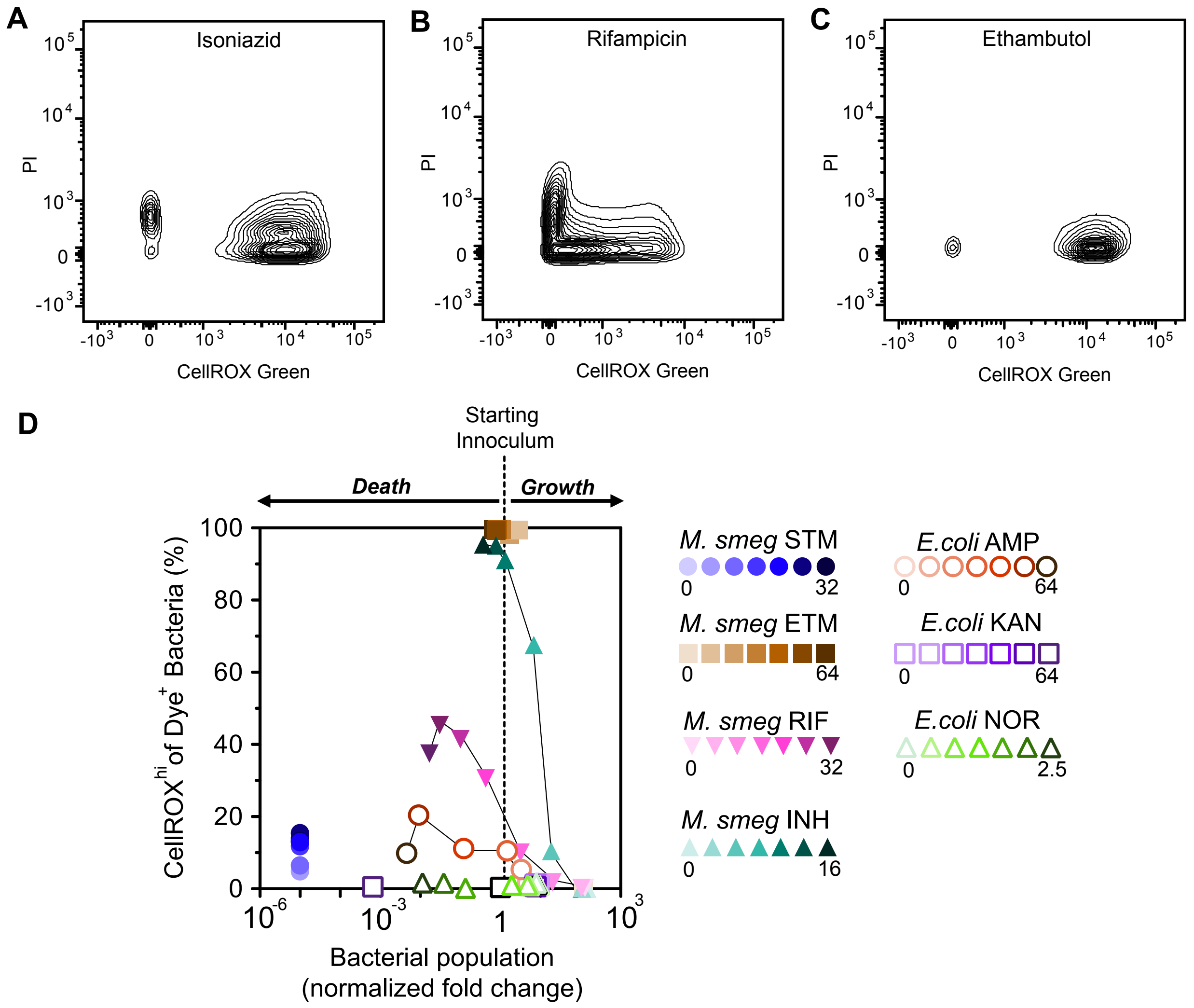

Supplementary Figure S4. Characteristics of *M. smegmatis* and *E. coli* under antibiotic stress. (A-C) Representative contour plots of *M. smegmatis* co-stained with PI and CellROX Green after 24 h antibiotic treatment. **(D)** ROS (CellROX^{hi}) proportion of population plotted versus CFU for different antibiotic treatments in *M. smegmatis* (24 h treatment) or *E. coli* DH5a (1 h treatment). Although dose-dependent killing and ROS generation does occur for some antibiotics, it is not a commonality. Color intensity indicates increasing concentration of antibiotic in µg/mL.

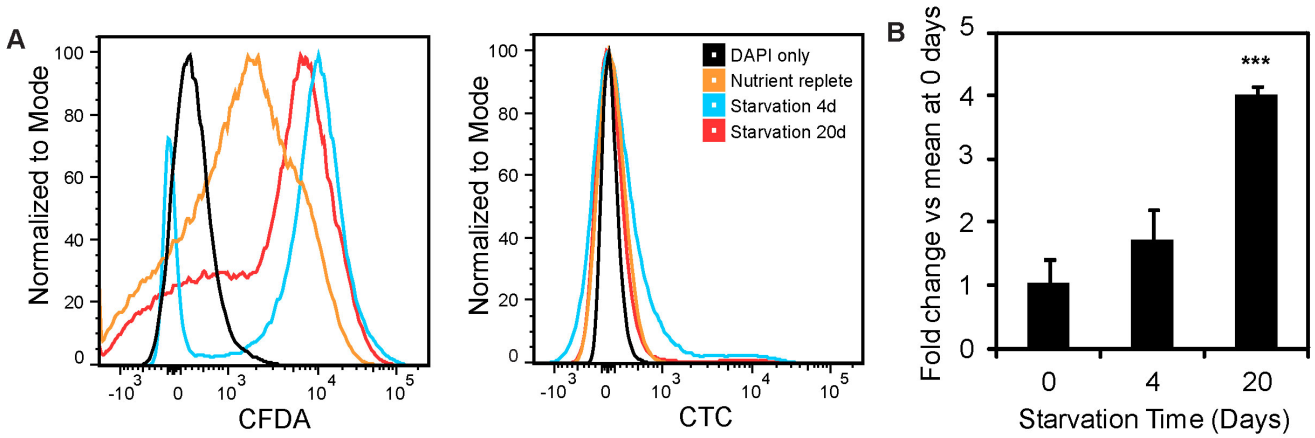

Supplementary Figure S5. Increased esterase and respiratory activities and up-regulation of BCG_1316c (rifampicin efflux pump) during starvation. (A) Histograms of DNA^+ (DAPI-stained) *M. bovis* BCG demonstrating increased CFDA (left) and CTC (right) fluorescence in starvation-induced non-replicating bacteria (starvation: incubation in PBS + 0.05% tyloxapol for 4 d or 20 d) compared to replicating bacteria in nutrient-replete (7H9) media. **(B)** Relative expression of BCG_1316c mRNA was determined by RNA-seq by counts of paired end reads. Data retrieved from GEO (study GSE66883). Mean ± SEM, N = 3. One-way ANOVA with Bonferroni multiple comparisons test, *** $P < 0.001$ (for starvation day = 20 versus starvation days = 0 and 4).
